# Porous Tantalum Implant in Treating Osteonecrosis of the Femoral Head: *Still a Viable Option*?

**DOI:** 10.1038/srep28227

**Published:** 2016-06-21

**Authors:** Jinhui Ma, Wei Sun, Fuqiang Gao, Wanshou Guo, Yunting Wang, Zirong Li

**Affiliations:** 1 Peking University China-Japan Friendship School of Clinical Medicine, 2 Yinghuadong Road, Chaoyang District, Beijing, 100029 China; 2 Center for Osteonecrosis and Joint Preserving & Reconstruction, Department of Orthopaedic Surgery, China-Japan Friendship Hospital, 2 Yinghuadong Road, Chaoyang District, Beijing, 100029 China

## Abstract

The purpose of this study is to evaluate the survivorship and risk factors for radiographic progression and conversion to total hip arthroplasty (THA) after porous tantalum implant surgery in the treatment of osteonecrosis of the femoral head (ONFH). The study comprised 90 ONFH patients (104 consecutive hips) who were treated with a porous tantalum implant combined with bone grafting between June 2008 and December 2013. The patients were 19–61 years of age (mean age, 38 years). The mean follow-up was 42 months. The outcome measures included Harris hip score (HHS), radiographic outcome measures, and survivorship analysis with conversion to THA as the endpoint. The mean postoperative HHS was significantly lower than the mean preoperative HHS (P < 0.001). The Cox proportional hazards model showed that age and Association Research Circulation Osseous (ARCO) stage were independent risk factors for conversion to THA, while age, China-Japan Friendship Hospital (CJFH) type, and ARCO stage were independent risk factors for radiological progression. Ultimately, only 52.9% hips survived. Porous tantalum implant surgery combined with bone grafting is not a viable option for treating ONFH, especially in patients >35 years of age with preoperative ARCO stage III and CJFH type L3.

Osteonecrosis of the femoral head (ONFH) is one of the most common refractory diseases in orthopedics. ONFH is a progressive disease that often occurs in young adults^1^. The natural history of ONFH generally leads to collapse of the femoral head, and if effective treatment methods are not used, the hip joint deteriorates and undergoes degenerative changes^2^,^3^. ONFH may be idiopathic or secondary to numerous diseases. Some spontaneous occurrences lack an obvious etiology, whereas most cases occur secondary to trauma^4^. Non-traumatic ONFH has been associated with corticosteroid usage, alcoholism, infection, hyperbaric events, storage disorders, marrow infiltrating diseases, coagulation defects, immoderately low or high temperatures, and some autoimmune diseases^4^,^5^. In trauma, the normal vascular supply to the femoral head is damaged, leading to ONFH.

The pathogenesis of non-traumatic ONFH is unclear, but it may be attributed to vascular injury, altered lipid metabolism/fat emboli, cell and bone death, mechanical stress, elevated intracortical pressure, and defective bone repair^4^,^6^. When examining the bone structure of the femoral head in patients with ONFH under the microscope, Kamal D *et al*. found vast areas of fibrosis, narrow bone trabeculae, obstructed or clotted blood vessels, hypertrophic fat cells, bone sequestration but also small cells and pyknotic nuclei^7^. Regardless of the etiology and mechanisms involved in the development of ONFH, a variety of trials have aimed to restore the mechanical structure and reconstruct the blood supply of the femoral head to prevent collapse. However, the treatment methods of ONFH remain controversial.

ONFH treatment aims to preserve the hip joint and delay hip arthroplasty. Although early diagnosis has been facilitated by the use of magnetic resonance imaging (MRI), there is still no standardized ONFH treatment protocol. An appropriate treatment strategy should be established that considers stage, type, lesion size, age, and joint function^8^. ONFH can be treated non-surgically or surgically, and each has its own potential complications. Non-surgical treatments^9^ include weight bearing with protection, drug treatment (nonsteroidal anti-inflammatory drugs, low molecular weight heparin, and vasodilator drugs), physical therapy (extracorporeal shock wave and high-frequency magnetic field therapy). The effect of these conservative treatments is not definite^10^. Several joint-preserving surgeries, such as core decompression, various osteotomy surgeries, lightbulb surgery, and non-vascularized or vascularized fibular grafting can also be implemented^11^,^12^,^13^,^14^. However, most studies reported less than satisfactory surgical outcomes^15^.

Another joint-preserving surgery, in which a porous tantalum with good biocompatibility and elastic modulus that provides strong support to the subchondral plate is implanted, provides an additional ONFH treatment option^16^. However, the reported results to date have varied considerably^17^,^18^,^19^,^20^, and its long-term clinical efficiency remains unclear despite its advantages. Meanwhile, there are some disadvantages of porous tantalum implantation surgery alone. A tantalum implant with a 10-mm diameter cannot provide sufficient mechanical support for the subchondral bone. Besides, the tantalum implant is associated with little bone ingrowth into the necrotic area^17^,^21^. Liu B *et al*.^22^ demonstrated that combining a tantalum implant with bone grafting displayed a promising short-term clinical outcome for patients with early-stage ONFH. Thus, to provide more structural support to the subchondral bone and enable better ingrowth of new bone tissue into the grafted bone and tantalum implant, here we combined porous tantalum implantation with bone grafting to treat patients with ONFH and evaluated their clinical and radiological outcomes.

## Materials and Methods

### Study population

A total of 101 patients with non-traumatic ONFH (117 consecutive hips) who underwent porous tantalum implant surgery in combination with bone grafting between June 2008 and December 2013 were included in this retrospective study. The patients were diagnosed with ONFH based on clinical history, physical examination, and radiological evaluations (X-ray and MRI) by orthopedic surgeons in our department. The inclusion criterion was a diagnosis of non-traumatic ONFH (ARCO stage II or III). The exclusion criteria were skin damage in the surgical region, active infection of the affected hip, clotting disorder, anemia (hemoglobin <100 g, white blood cell count <4 × 10^9^), secondary arthritis (ARCO stage IV), or having received any other type of surgical treatment. The study was approved by the Institutional Review Board on Human Studies of the Ethical Committee of China-Japan Friendship Hospital (CJFH), and the methods were performed in accordance with the Declaration of Helsinki. Written informed consent was obtained from all subjects allowing us to store their data in our hospital database and use it for clinical research.

**Figure 1:**
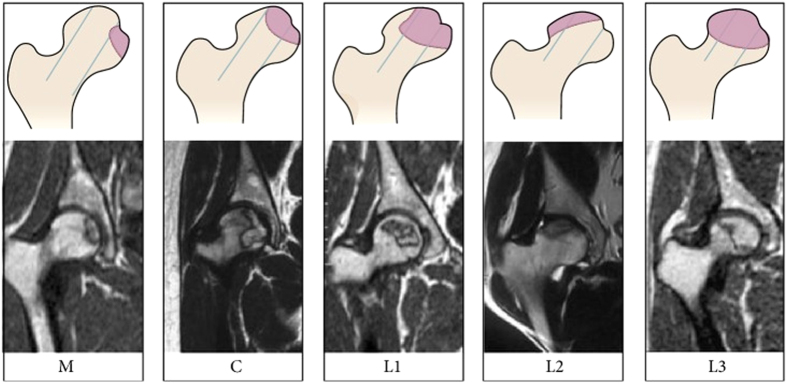
Schematic diagram and magnetic resonance image of China-Japan Friendship Hospital (CJFH) classification for osteonecrosis of the femoral head based on three pillars^19^. Type M: necrosis involves the medial pillar. Type C: necrosis involves the medial and central pillars. Type L1: necrosis involves the three pillars but the partial lateral pillar was preserved. Type L2: necrosis involves the entire lateral pillar and part of the central pillar. Type L3: necrosis involves the three pillars including the cortical bone and marrow.

The patients were evaluated preoperatively both clinically and radiologically using the Harris hip score (HHS)^23^, CJFH type (Fig. 1)^24^, and the ARCO classification system^25^. According to ARCO stage, 49 hips had stage II disease and 68 hips had stage III disease. CJFH types were as follows: L1, 15 hips; L2, 59 hips; L3, 40 hips; M, 0 hips; and C, three hips. Eleven patients (13 hips) were lost during follow-up for multiple reasons. Thus, a total of 90 patients (104 consecutive hips) were studied in the data analysis (Table 1), including 14 with bilaterally affected hips and 76 with unilaterally affected hips. The mean patient age was 38.48 ± 8.14 years (range, 19–61 years), of which 66 were male and 24 were female. The average body mass index (BMI) was 25.04 ± 3.32 kg/m^2^. ONFH was idiopathic in 14 hips, secondary to steroid use in 50 hips, and associated with alcohol use in 40 hips. Mean follow-up time was 42.96 ± 18.71 months (range, 1–78 months).

### Staging and typing

**Figure 2:**
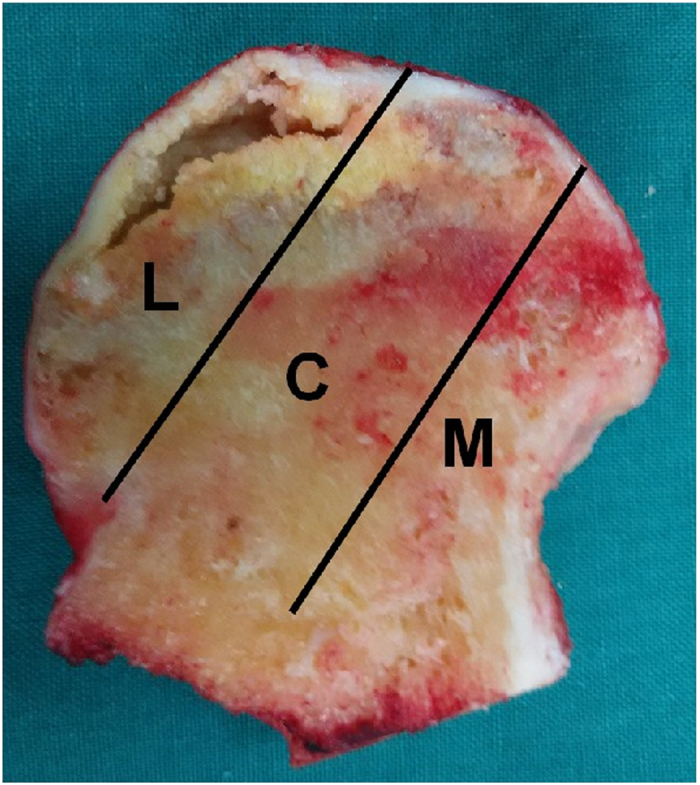
Image of coronal section of the femoral head showing three pillars of the femoral head: lateral (30%), central (40%), and medial (30%)^23^

The preoperative stages by ARCO classification system were stage II in 42 hips and stage III in 62 hips. All subjects underwent an MRI evaluation according to CJFH type^22^ (Fig. 1) for ONFH based on three pillars (Fig. 2)^26^. According to the involvement of necrosis in the three pillars on a mid-coronal section on MRI, ONFH location was divided into three types (M, C, and L), and the intact degree of the lateral pillar was divided into subtypes (L1, L2, and L3). Using this type to evaluate the efficacy of tantalum implant surgery for ONFH, the preoperative CJFH types were type L1 in 13 hips, type L2 in 53 hips, and type L3 in 38 hips.

### Surgical procedure

All surgeons using this device had prior surgical experience and completed their learning curve during the study period. Before surgery, the patients were placed in the supine position on an orthopedic traction table. The affected hip was placed neutrally in an adducted position. Under fluoroscopic guidance, a guide pin was drilled from the proximal lateral femur into the anterolateral necrotic area of the femoral head. A core reamer was placed over the guide pin to create a 10-mm-diameter bone channel through which necrotic bone tissue and the surrounding hardened area was scraped off by a long curette. Autologous bone curetted from the ipsilateral iliac bone was then deposited into the clean necrotic area through the bone channel. A measured porous tantalum implant (Zimmer, Warsaw, IN, USA) with an 85-mm length and 10-mm diameter was inserted under fluoroscopic guidance until it abutted the subchondral plate.

### Postoperative management and rehabilitation

A prophylactic antibiotic (cefuroxime sodium 2.25 g bid) was intravenously used for the first 24 hours after surgery to prevent a wound infection. The patients were instructed to be non-weight-bearing for 6 weeks, after which point partial weight-bearing with a walking aid was allowed for the following 6 weeks. All patients were allowed to perform full-weight-bearing walking 12 weeks after the procedure. Patients who had severe pain and or limited functioning following tantalum implant surgery were identified by the surgeons as requiring conversion to THA.

### Outcome assessment

The postoperative clinical and radiological evaluations were based on HHS and plain radiographs. The endpoint was defined as conversion to THA. Radiographic failure was defined as femoral head collapse progressing past ARCO stage III or from ARCO stage II to ARCO stage III or joint space narrowing. The radiological progression was independently determined by two observers (J.M. and F.G.). In the case of disagreement, all authors discussed the details until consensus was reached. The last follow-up was determined by the time taken for THA conversion or the longest time of hip survival. Complications from the surgical procedure were closely monitored and recorded for each patient.

### Statistical Analysis

The data were analyzed using SPSS version 19.0 statistical software (SPSS Inc., Chicago, IL, USA). Measurement data are reported as mean ± standard deviation. Statistical differences in survival rate were calculated using log-rank analysis of Kaplan-Meier survival curves with the end point of conversion to THA. The Wilcoxon signed-rank test was used to compare pre- and postoperative HHS. The Wilcoxon-Mann-Whitney test was used to compare HHS decline by stage and age. The Wilcoxon-Kruskal-Wallis test was used to compare HHS among different type and etiology groups. A chi-square test was performed to compare the rate of radiological progression among the different stages, types, and age groups. We used the Cox proportional hazards model to analyze the independent factors associated with conversion to THA and radiological failure. All tests were two-tailed at the 5% level of significance.

## Results

### Harris hip score

The mean postoperative HHS for all hips (at a mean 69.27 ± 14.63 points) was less than the mean preoperative HHS (at a mean 76.25 ± 12.72 points) at the last follow-up or before THA conversion (P < 0.001). The mean preoperative HHS of hips after clinical failure was 77.61 ± 9.59, while that of hips that survived was 75.04 ± 14.96, which is not significantly different (P = 0.992). The mean HHS decline was 6.98 ± 18.08. The mean HHS decline among hips in the different age groups, ARCO stages, and CJFH types are shown in Table 2. Despite the different ONFH etiologies (steroid use, excessive alcohol intake, or idiopathic origin), there were no significant differences between cases with respect to preoperative HHS (P = 0.666) or postoperative HHS (P = 0.175).

### Conversion to THA

Overall, 49 hips (47.1%) were converted to THA. The mean patient age of these cases was 40.7 years, while the average BMI was 25.1 kg/m^2^. Table 3 summarizes the analytical results and clinical characteristics of the hips converted to THA. The average time from the porous tantalum implant surgery to conversion to THA was 29.65 months (range, 1–60 months). The average age at tantalum implant surgery in patients converted to THA was 40.65 ± 7.16 years versus 36.55 ± 8.53 years in patients who were not. This difference is statistically significant (P = 0.010).

**Figure 3:**
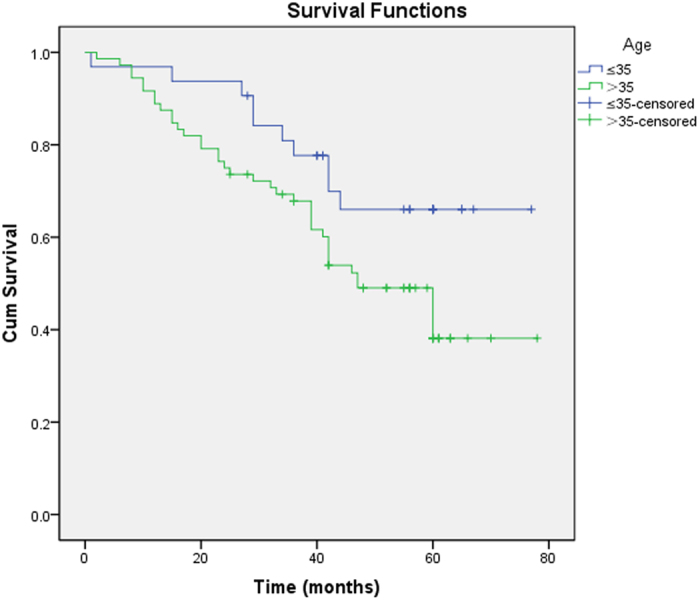
Comparison of survival time between age groups ( ≤ 35 years and > 35 years). The survival time was significantly shorter in the latter group (P = 0.034).

**Figure 4:**
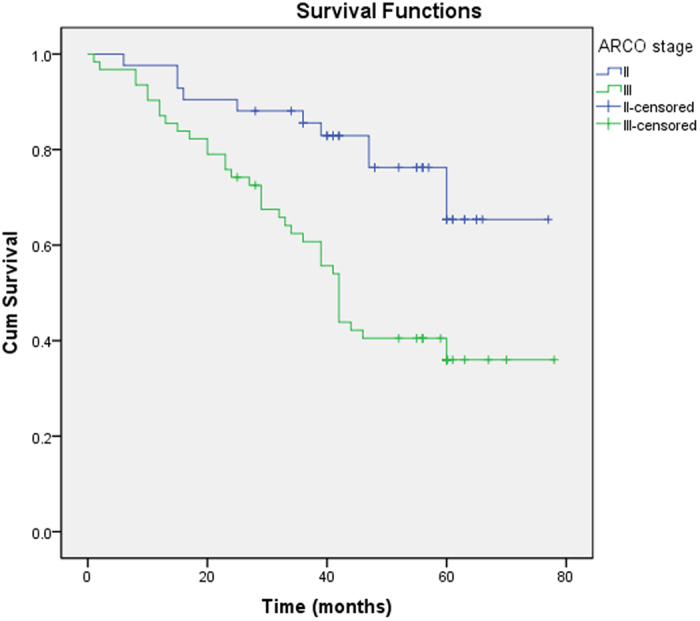
The survival time between Association Research Circulation Osseous (ARCO) stages II and III. The survival time was significantly shorter in the latter (P = 0.001).

**Figure 5:**
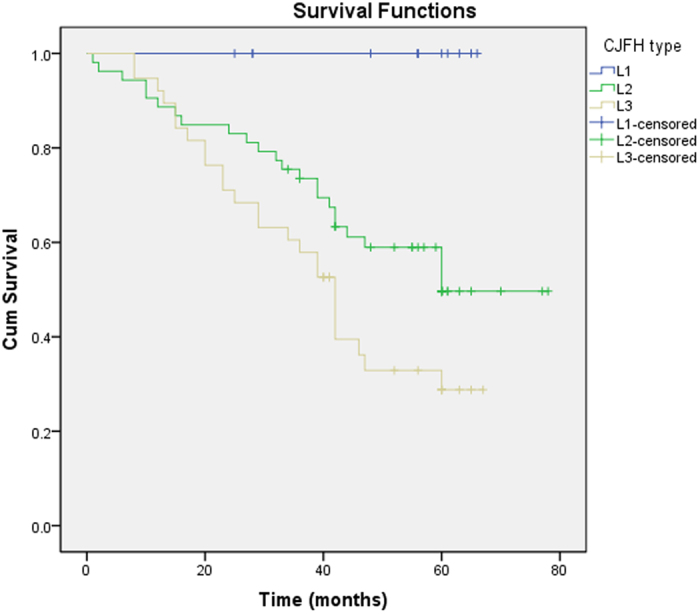
The survival time among different China-Japan Friendship Hospital (CJFH) classifications. The survival time of CJFH type L3 hips was significantly shorter than that of CJFH type L1 (P < 0.001) or L2 (P = 0.040) hips.

Most of the patients converted to THA had ARCO stage III and CJFH type L3 and were >35 years of age. Survival rates of the different groups were analyzed using Kaplan-Meier analysis (Table 2). Hips were more prone to failure in patients >35 years old (P = 0.034) (Fig. 3). The survival time of hips with an ARCO stage III was significantly shorter than that of hips with an ARCO stage II (P = 0.001) (Fig. 4). The survival time of CJFH type L3 hips was significantly shorter than that of CJFH type L1 (P < 0.001) or L2 (P = 0.040) hips, while the survival time of CJFH type L2 hips was significantly shorter than that of hips with CJFH type L1 hips (P = 0.010) (Fig. 5).

However, no significant difference was found in survivorship curves when stratified by sex (P = 0.244), bilateral disease (P = 0.802), BMI ≧ 25 kg/m^2^ (P = 0.515), bone marrow edema (P = 0.332), preoperative HHS ≧ 80 (P = 0.078), or etiology (P = 0.589) (Table 3).

### Radiographic assessment

**Figure 6:**
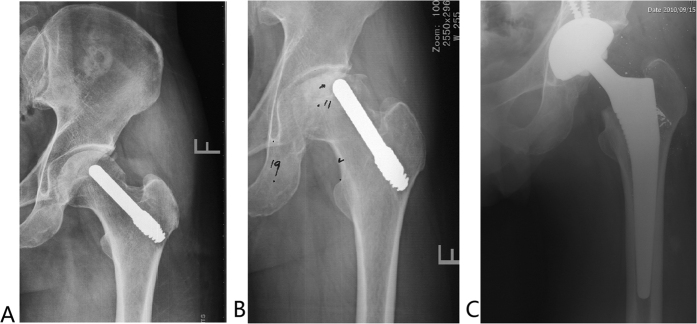
Anteroposterior radiograph of a 49-year-old man with Association Research Circulation Osseous (ARCO) stage II osteonecrosis of the left hip undergoing a tantalum porous implantation was taken intraoperatively (**A**) and 14 months postoperatively (**B**), at which time the collapse had progressed and the joint space had narrowed. The patient underwent total hip arthroplasty 14 months after tantalum porous implantation (**C**).

**Figure 7:**
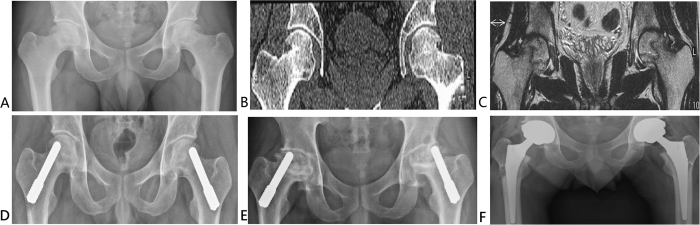
Anteroposterior X-ray and computed tomography examination of a representative case of 29-year-old man with bilateral Association Research Circulation Osseous stage III osteonecrosis of the femoral head (ONFH) (**A,B**). Preoperative magnetic resonance image (coronal section, T1WI) of the patient showing bilateral necrosis of the bone marrow and the cortical bone. CJFH type is based on the three pillars. The bilateral sides are both type L3. Necrosis involves all three pillars (**C**). Porous tantalum implants were inserted in the bilateral hips to treat ONFH (**D**). Anteroposterior radiographic films showing that the bilateral hips progressed collapsed from ARCO stage III to ARCO stage IV 42 months after a porous tantalum implantation surgery (**E**). Two porous tantalum implants inserted in the bilateral hips required conversion to THA after 42 months (**F**).

On the whole, 55 of 104 hips (52.88%) were classified as radiographic progression (Figs 6 and 7). The rate of radiological progression of ARCO II stage hips was 38.10% (16/42), whereas the progression rate of ARCO III stage hips was 62.90% (39/62) (P = 0.013). By CJFH type, no significant difference in the rate of radiographic progression between type L1 hips (30.77%, 4/13) and type L2 hips (47.17%, 25/53) (P = 0.286) was observed. The rate of radiographic progression in type L3 hips (68.42%, 26/38) was higher than that of types L1 (P = 0.017) and L2 (P = 0.044). The rate of radiographic progression in patients ≤ 35 years was 34.38% (11/32) versus 61.11% (44/72) in patients >35 years (P = 0.012).

Cox proportional-hazards analysis (Tables 4 and 5) revealed that age (P = 0.029; hazard ratio [HR], 0.397; 95% confidence interval [CI], 0.173–0.912) and ARCO stage (P = 0.008; HR, 0.361; 95% CI, 0.170–0.767) were independent risk factors for conversion to THA; age (P = 0.007; HR, 0.336; 95% CI, 0.152–0.743), CJFH type (P = 0.004), and ARCO stage (P = 0.028; HR, 0.474; 95% CI, 0.243–0.924) were independent risk factors for radiological progression. While conversion to THA and radiological failure were not correlated with sex, BMI ≥ 25 kg/m^2^, bilateral hip involvement, preoperative HHS ≥80, ONFH etiology, and bone marrow edema.

### Complications

Two patients developed a postoperative infection (*Staphylococcus epidermidis*). The two patients were treated with one-stage tantalum implant extraction and the insertion of antibiotic-loaded balls of poly(methyl methacrylate) (PMMA) on a string. The patients were treated with antibiotics, followed by two-stage conversion to THA. None of them developed other postoperative complications such as a femoral neck or intertrochanteric fracture.

## Discussion

As a joint-preserving surgical method, porous tantalum implantation possesses some inherent advantages such as high porosity, good biocompatibility, excellent corrosion resistance, high friction tolerance, and a modulus of elasticity relative to that of bone^16^. The early results of a porous tantalum implant for the treatment of early-stage osteonecrosis are encouraging^17^. However, porous tantalum implants also have some shortcomings, as evidenced by several reports^20^,^21^,^27^ of clinical failure requiring conversion to THA.

Clinical failure is prone to occur in patients with a large degree of necrosis in the femoral head. Porous tantalum implants are only 10 mm in diameter, a size that can only support the subchondral plate on a small localized area that is insufficient in cases of large necrotic lesions. Also, no new bone formation or vascular ingrowths were found in large necrotic portions of the femoral head. Collapse of the femoral head then occurs in the area without tantalum implant structural support^21^. Our study showed that patients with CJFH type L3 hips were more prone to clinical failure than those with type L1 or L2. The patients with CJFH type L3 hips and large necrotic areas involving all the three pillars of the femoral head had poor prognosis and the survival rate of CJFH type L2 hips was significantly lower than that of type L1 hips. This illustrates that preservation of the lateral pillar is highly important in porous tantalum implantation prognosis.

Motomura *et al*.^28^ demonstrated that the lateral pillar of the necrotic lesion appeared to collapse first and that larger lesion size was likely to aggravate collapse extent. The efficacy of joint-preserving surgery for patients with ONFH involving the lateral pillar was unsatisfactory^29^. Another study^22^ showed that clinical outcomes of porous tantalum implants for treating ONFH were associated with necrotic lesion type. They observed that the survival time was significantly shorter in patients with necrotic lesions involving the lateral column. Liu *et al*.^30^ demonstrated that femoral heads with large necrotic lesions might benefit less from the mechanical support offered by porous tantalum implantation. Another study^27^ indicated that the relative risk of patients with large necrotic lesion requiring conversion to THA after porous tantalum implantation for treating ONFH was 3.69 times higher than those without large necrotic lesions. Our results were similar to their findings^22^,^27^,^30^. Therefore, a porous tantalum implant should be carefully used in patients with large necrotic lesions, especially CJFH type L3 involving the lateral pillar.

Our study results differ from those involving encouraging outcomes of porous tantalum implantation for treating ONFH described by Tsao *et al*.^17^ and Shuler *et al*.^18^. A possible reason our inferior worse results may be the inclusion of patients with subchondral collapse (ARCO stage III) with or without flattening. Collapse of the femoral head is a turning point in the course of femoral head necrosis^31^. Once the femoral head collapses ( > 2 mm), hip conservation procedures become ineffective and THA remains the only option^8^. Veillette *et al*.^19^ suggested that the effect of a porous tantalum implant in treating post-collapse hips was not ideal. Another study by Nadeau *et al*.^20^ showed that the overall success rate at the final follow-up (mean 23.2 months) was 44.5% and that failures (10/18 hips, 55.6%) occurred at a mean time of 11.7 months. Their result was higher than our overall failure rate of 47.1% (26.2% ARCO stage II, 61.3% ARCO stage III), and this is likely due to the fact that all of their hips were Steinberg stage III and IV ONFH. Our results revealed that the survival time of ARCO stage III hips was significantly shorter than that of ARCO stage II hips, and the mean postoperative HHS in patients with ARCO stage III hips was significantly lower than that in patients with ARCO stage II hips. Besides, the conclusion of a meta-analysis^32^ was that the clinical outcome of joint-preserving surgeries for post-collapse hips was not encouraging. Another study^33^ implied that the failure rate was 56.5% (13/23 ARCO stage I and II hips) after porous tantalum implantation after a mean follow-up of 1.45 years, and their outcomes compared with core decompression alone revealed that the porous tantalum implant did not show superior results, while the procedure was associated with increased costs and a prolonged operation time. Thus, their study did not recommend porous tantalum implantation for the treatment of early-stage ONFH. Our sample size is larger and follow-up is longer than those of previous studies^20^,^33^. However, the study by Liu *et al*.^27^ revealed that modified porous tantalum implantation to treat patients with Steinberg stages I-IVA ONFH obtained encouraging survival rates and delayed or prevented THA, while another study by Varitimidis *et al*.^34^ reported positive overall results of tantalum rod implantation for the treatment of pre- and post-collapse ONFH. However, no uniform recommendation for this procedure in the treatment of ONFH exists. Meanwhile, THA is not suitable for the young active patients with ONFH because most of them will likely outlive their prosthesis and require revision THA^20^. Besides, several studies^35^,^36^ have reported complications of primary THA in young patient populations. The primary goal of our study is to preserve the hip joint and delay THA using a modified approach of porous tantalum implant combined with bone grafting for young patients with early post-collapse ONFH who were reluctant to undergo THA. However, this procedure did not show satisfactory clinical outcomes for post-collapse patients.

The prognosis of ONFH patients often correlates with age, sex, etiology, and preoperative stage^37^. Patients > 35 years are more prone to ONFH^38^. As such, we divided the ONFH patients into those ≤ 35 years old and those > 35 years old. Our Cox hazard model analysis revealed that age and preoperative ARCO stage are the major risk factors of conversion to THA. The patients > 35 years old had poorer prognosis than those ≤ 35 years old, which led us to conclude that the former are more prone to clinical failure. Our results are consistent with Nadeau’s report^20^ in which the mean age at tantalum implantation in cases of failure was 50.1 years versus 36.8 in cases of success. In our study, the average age at tantalum implantation in patients who required THA was 40.65 years old compared to 36.55 in patients who did not. He suggested that younger patients undergoing tantalum implantation could obtain satisfactory outcomes. This indicates that age is one of the prognostic factors of porous tantalum implantation.

Several earlier studies suggested that bone marrow edema is a poor prognostic factor in patients with ONFH^38^,^39^. Liu *et al*.^27^ revealed that the survival rate of hips with bone marrow edema in ONFH patients treated with a porous tantalum implant is significantly lower (65.34% at 60 months) than in patients without bone marrow edema (85.71% at 60 months). They identified that bone marrow edema is the independent predictor of conversion to THA irrespective of disease stage. Here we did not discover bone marrow edema as the independent prognostic factor for clinical failure or radiographic progression. However, further large-scale studies are needed to illustrate the correlation between bone marrow edema and the prognosis of porous tantalum implantation for treating ONFH.

One limitation of our study is that all patients were treated with a porous tantalum implant in combination with autologous iliac bone grafting. Another is that we did not analyze whether autologous bone grafting influenced the prognosis of porous tantalum implants. Additionally, we have no histological evidence that the bone ingrowths into porous tantalum implants is paucity. Further studies with large samples are needed to elucidate the long-term clinical outcomes of a porous tantalum implant combined with bone grafting in the treatment of ONFH.

Overall, only 52.9% hips achieved acceptable results; thus, a porous tantalum implant combined with bone grafting surgery does not seem to be a viable option for treating ONFH. Procedural failure is associated with several factors: (1) large necrotic portion of the femoral head as well as involvement of the three pillars (e.g. CJFH type L3); (2) a 10-mm-diameter porous tantalum implant cannot provide enough mechanical support; (3) patients who are > 35 years old and have a preoperative ARCO stage III (subchondral collapse) are more prone to clinical failure; and (4) the porous tantalum implant must be removed in cases of deep infection. Thus, our study findings do not support the insertion of a porous tantalum implant combined with bone grafting for treating ONFH. The determination of patients who are more likely to benefit from this treatment and the selection of the best treatment modality for different ONFH patients will be done in future studies.

## Additional Information

**How to cite this article**: Ma, J. *et al*. Porous Tantalum Implant in Treating Osteonecrosis of the Femoral Head: *Still a Viable Option*? *Sci. Rep.* **6**, 28227; doi: 10.1038/srep28227 (2016).

## Figures and Tables

**Table 1:** Preoperative Patient Demographics.

Demographic	No.
Patients (M/F)	90
Male	66
Female	24
Mean age (range), y	38.48 ± 8.14(19–61)
Invasive hip	
Unilateral	76
Bilateral	14
Etiology	
Idiopathic	14
Corticosteroids	50
Alcohol	40
ARCO stage	
Stage II	42
Stage III	62
CJFH classification	
L1	13
L2	53
L3	38

**Table 2:** Harris score and survival time.

	Group	HHS decline	p value	Survival rate	Survival time (M)	p value
age	age > 35 years	2.24 ± 25.13	P = 0.158	33/72 (45.82%)	40.74	P = 0.034
	age ≤35 years	9.09 ± 13.56		22/32 (68.75%)	47.97	
stage	II	5.61 ± 12.38	P = 0.166	31/42 (73.81%)	47.50	P = 0.001
	III	7.92 ± 21.14		24/62 (38.71%)	39.89	
CJFH stype	L1	−2.87 ± 15.16	P = 0.011	13/13 (100%)	51.38	P = 0.001
	L2	5.61 ± 17.03		29/53 (54.72%)	44.70	
	L3	12.27 ± 19.05		13/38 (34.21%)	37.66	

**Table 3:** The analytic results and clinical characteristics of hips converting to THA.

Index	Number of hips	Hips converting to THA	Survival rate at 3-year (SE)	Survival rate at 5-year (SE)	P value (Log-rank test)
*n*	104	49	70.8% (4.5%)	47.5% (5.5%)	
Gender (M/F)	77/27	34/15	76.4% (4.9%)/54.9% (6.2%)	48.9% (6.6%)/42.2% (9.8%)	0.244
BMI ≧ 25 kg/m^2^ (Yes/No)	49/55	22/27	71.2% (6.5%)/70.4% (6.2%)	49.5% (8.0%)/45.1% (7.3%)	0.515
Invasive hip (uni/bi)	76/28	42/7	68.5% (4.9%)/85.7% (9.4%)	48.2% (5.9%)/44.5% (14.3%)	0.802
bone marrow edema (Yes/No)	54/50	24/25	72.2% (6.1%)/69.6% (6.6%)	54.8% (6.9%)/35.8% (8.9%)	0.332
preoperative HHS ≧ 80 scores (Yes/No)	48/56	18/31	80.6% (5.8%)/62.4% (6.5%)	52.9% (8.9%)/42.7% (6.9%)	0.078
etiology					0.589
corticosteroids	50	26	67.8% (6.6%)	45.5% (7.3%)	
alcohol	40	17	79.7% (6.4%)	46.3% (9.7%)	
idiopathic	14	6	57.1% (13.2%)	57.1% (13.2%)	
Age (years)					0.034
35	72	39	67.8% (5.5%)	38.1% (6.8%)	
≤35	32	10	77.7% (7.4%)	66.0% (8.9%)	
ARCO stage					0.001
Stage II	42	11	85.6% (5.5%)	65.4% (9.3%)	
Stage III	62	38	60.7% (6.3%)	36.0% (6.4%)	
CJFH type					0.001
L1	13	0			
L2	53	24	73.5% (6.1%)	49.6% (7.6%)	
L3	38	25	57.9% (8.0%)	28.8% (8.1%)	

**Table 4:** The results of Cox proportional-hazards model for conversion to THA.

Variable selected	B	SE	Wald	P	HR	95% CI
Gender	−0.630	0.365	2.980	0.084	0.532	0.260~1.089
Age	−0.923	0.424	4.744	**0.029**	0.397	0.173~0.912
BMI	0.197	0.333	0.351	0.554	1.218	0.634~2.338
Bilateral hip involvement	0.112	0.429	0.068	0.794	1.119	0.483~2.591
Preoperative HHS	−0.293	0.338	0.752	0.386	0.746	0.385~1.447
Bone marrow edema	0.467	0.323	2.086	0.149	1.595	0.846~3.006
ONFH etiologies			0.527	0.768		
etiology (Corticosteroids)	0.311	0.496	0.393	0.531	1.365	0.516~3.606
etiology (Alcohol)	0.091	0.517	0.031	0.861	1.095	0.398~3.014
ARCO stage	−1.020	0.385	7.020	**0.008**	0.361	0.170~0.767
CJFH type			3.920	0.141		
type (L1)	−13.931	272.997	0.003	0.959	0.000	0.000~2.11E226
type (L2)	−0.615	0.310	3.918	0.048	0.541	0.294~0.994

B: regression coefficient; SE: standard error; HR: hazard ratio; Bold value indicate the significant P value.

**Table 5:** The results of Cox proportional-hazards model for radiological progression.

Variable selected	B	SE	Wald	P	HR	95% CI
Gender	−0.617	0.339	3.314	0.069	0.539	0.278~1.048
Age	−1.090	0.404	7.260	**0.007**	0.336	0.152~0.743
BMI	0.060	0.309	0.038	0.846	1.062	0.579~1.946
Bilateral hip involvement	0.027	0.402	0.004	0.947	0.539	0.443~2.140
Preoperative HHS	0.402	0.322	1.557	0.212	1.495	0.278~1.048
Bone marrow edema	0.465	0.311	2.226	0.136	1.591	0.864~2.930
ONFH etiologies			1.907	0.385		
etiology (Corticosteroids)	0.332	0.449	0.546	0.460	1.393	0.578~3.357
etiology (Alcohol)	−0.178	0.472	0.143	0.705	0.837	0.332~2.111
ARCO stage	−0.747	0.341	4.802	**0.028**	0.474	0.243~0.924
CJFH type			10.960	**0.004**		
type (L1)	−1.650	0.582	8.042	0.005	0.192	0.061~0.601
type (L2)	−0.733	0.303	5.846	0.016	0.481	0.265~0.870

B: regression coefficient; SE: standard error; HR: hazard ratio; Bold value indicate the significant P value.
